# Supplementary material for: Discovery of Novel Benzamide-Based Sigma-1 Receptor Agonists with Enhanced Selectivity and Safety
Source: Molecules. 2025 Sep 2;30(17):3584. doi: 10.3390/molecules30173584 (PMC12430443; doi:10.3390/molecules30173584)

Discovery of Novel Benzamide-Based Sigma-1 Receptor Agonists with Enhanced Selectivity and Preclinical Safety

Pascal Carato[1,†,‡], Bénédicte Oxombre[1,†], Séverine Ravez[1], Rajaa Boulahjar[1], Marion Donnier-Maréchal[1], Amélie Barczyk[2], Maxime Liberelle[1], Patrick Vermersch[1] and Patricia Melnyk [1,*]

[1] Univ. Lille, Inserm, CHU Lille, U1172—LilNCog—Lille Neuroscience & Cognition, F-59000 Lille, France
[2] Univ. Lille, Inserm, CHU Lille, U1286-INFINITE-Institute for Translational Research in Inflammation, Lille F-59000, France

***** Correspondence: patricia.melnyk@univ-lille.fr

[†] These authors contributed equally to this work

[‡] present address : Univ Poitiers, CIC INSERM 1402, F-86073 Poitiers, France

Figure S1. LC-MS and NMR spectra of compounds **2-7**

3-(Benzylmethylamino)-*N*-(3-chlorobenzyl)propanamide (2)

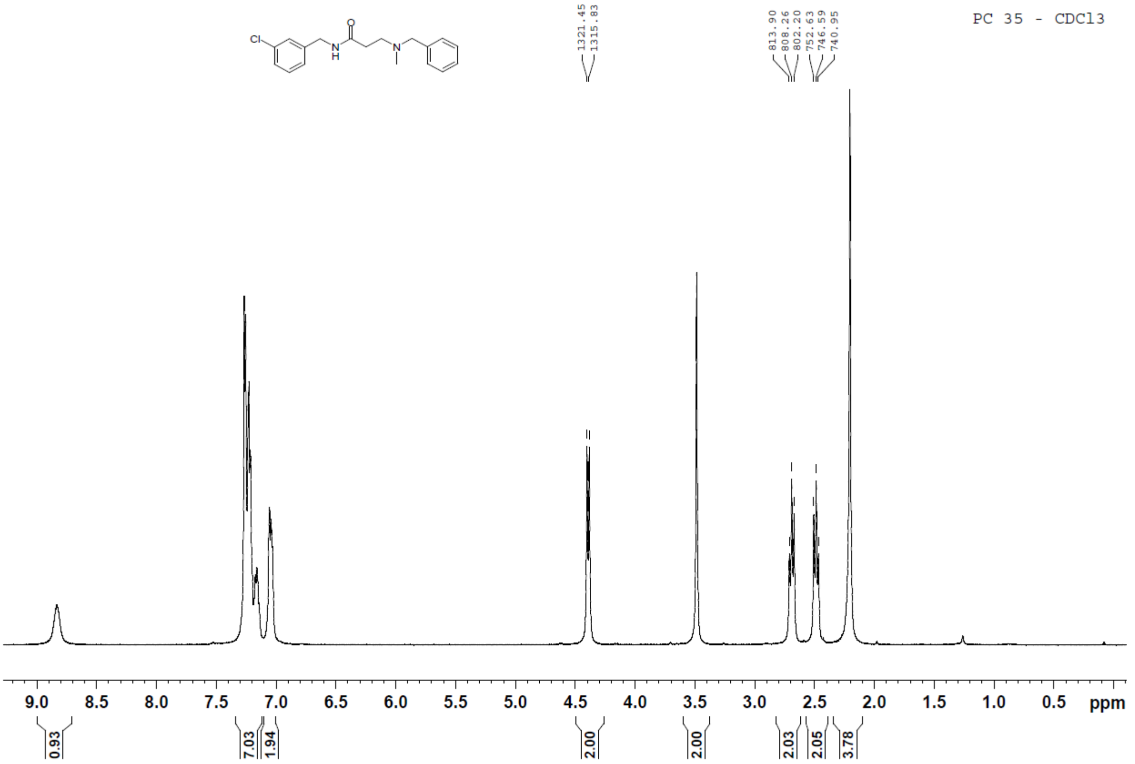

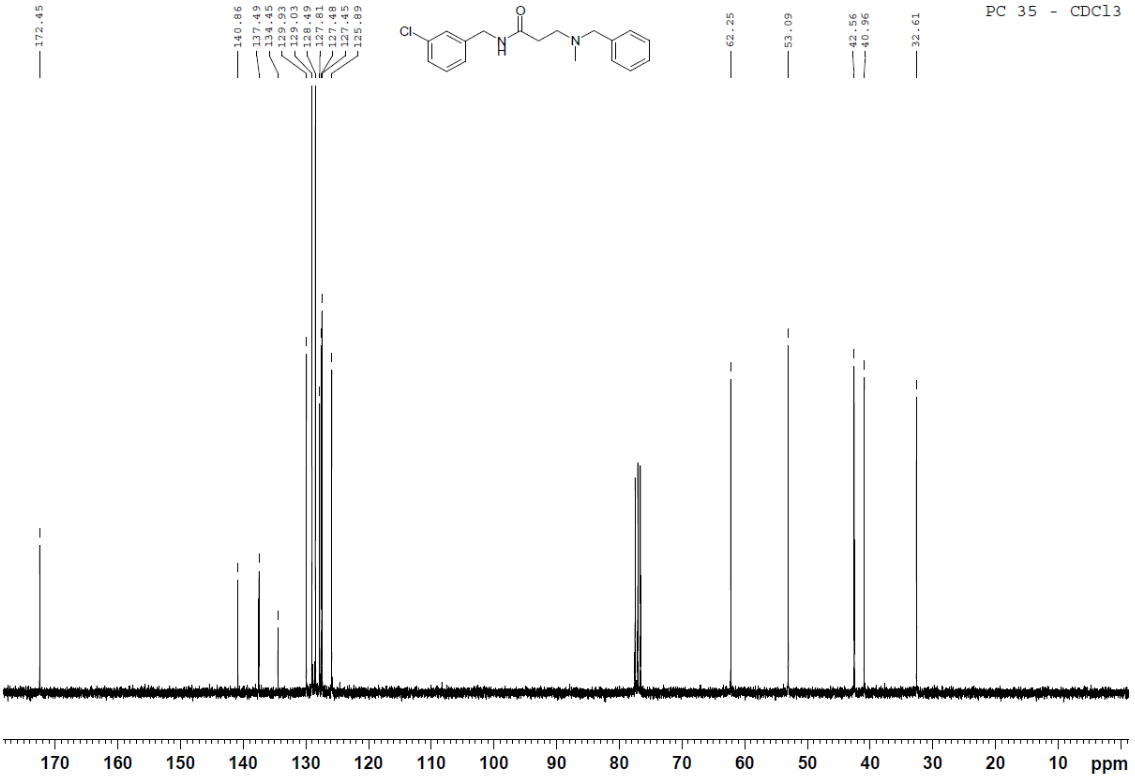

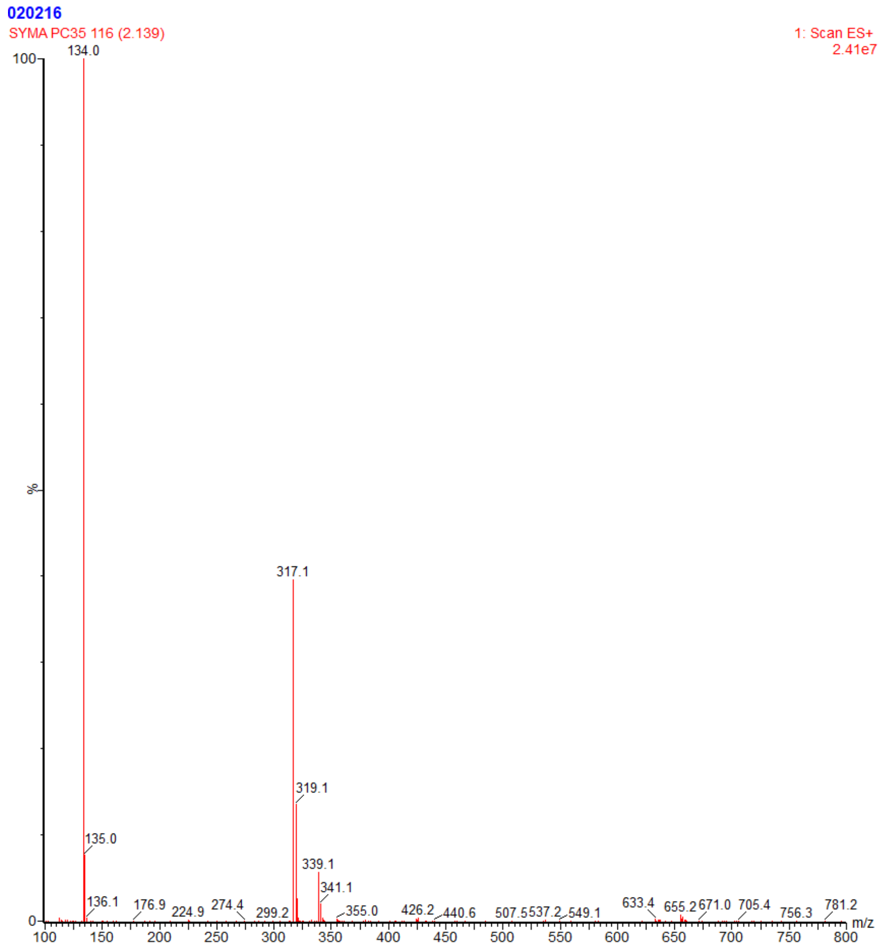
020216
SYMA PC35 116 (2.139)
1: Scan ES+
2.41e7
134.0
317.1
319.1
135.0
339.1
341.1
136.1
176.9
224.9
274.4
299.2
355.0
426.2
440.6
507.5
537.2
549.1
633.4
655.2
671.0
705.4
756.3
781.2
%
m/z
100
0

3-(Benzylmethylamino)-*N*-(4-chlorobenzyl)propanamide (3)

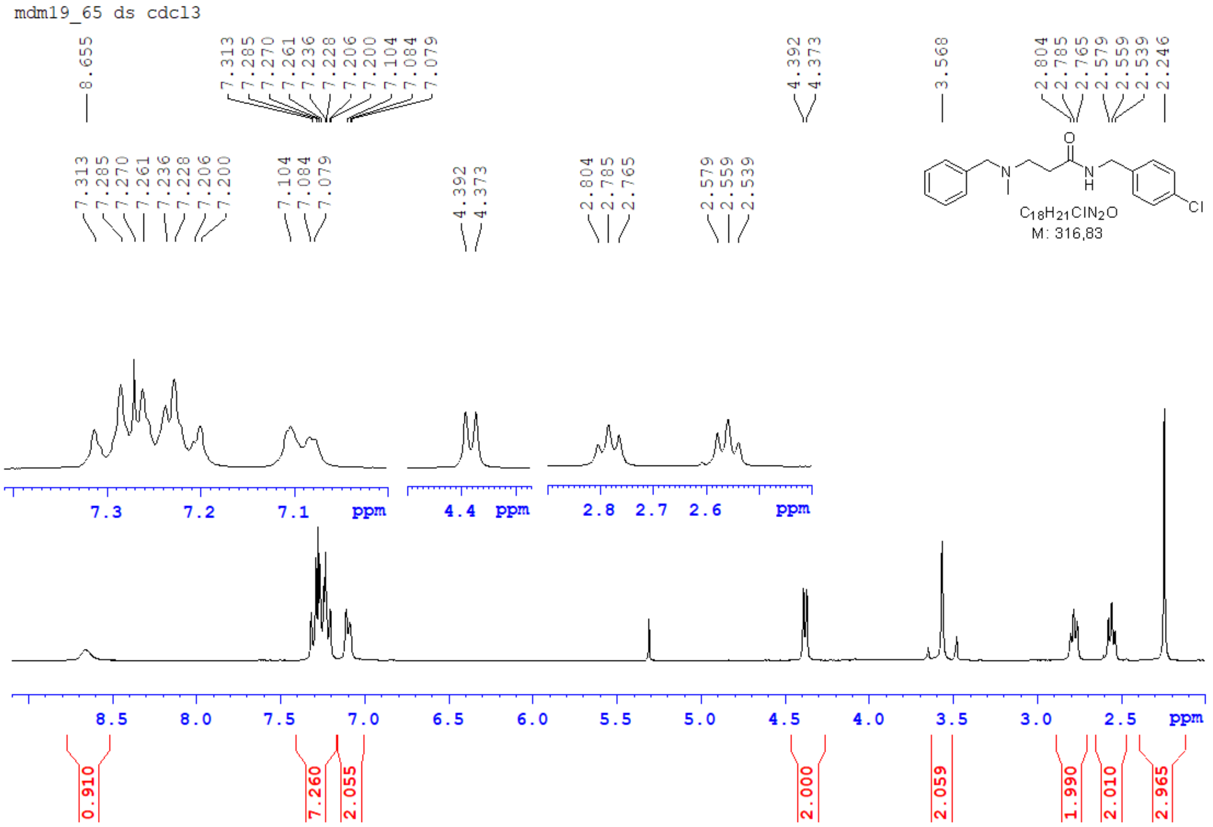

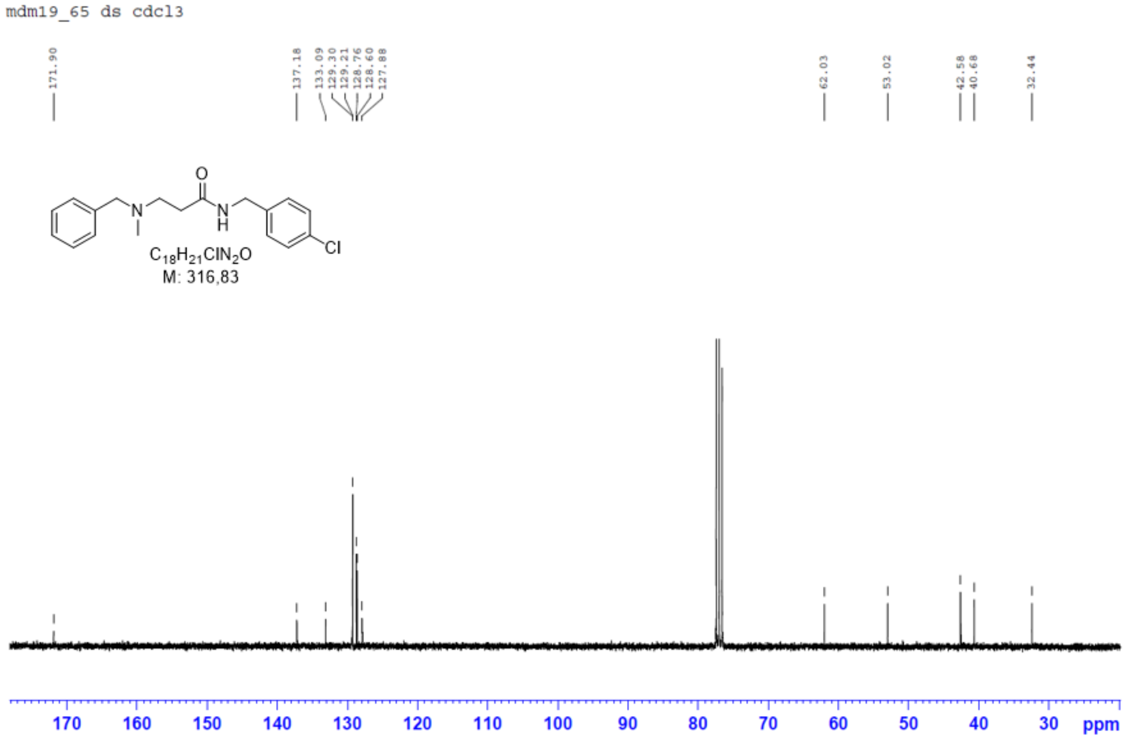

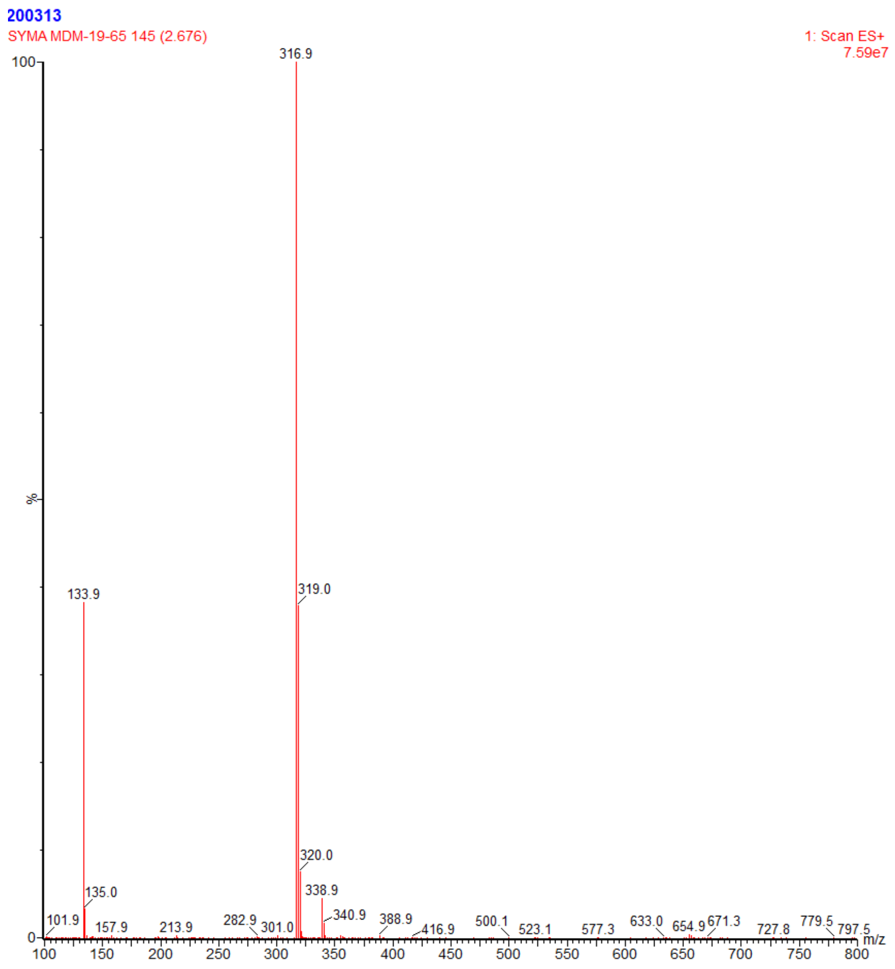
200313
SYMA MDM-19-65 145 (2.676)
1: Scan ES+
7.59e7
100
%
0
316.9
319.0
133.9
320.0
135.0
338.9
340.9
101.9
157.9
213.9
282.9
301.0
388.9
416.9
500.1
523.1
577.3
633.0
654.9
671.3
727.8
779.5
797.5
m/z
100
150
200
250
300
350
400
450
500
550
600
650
700
750
800

3-(Benzylmethylamino)-*N*-(3-bromobenzyl)propanamide (4)

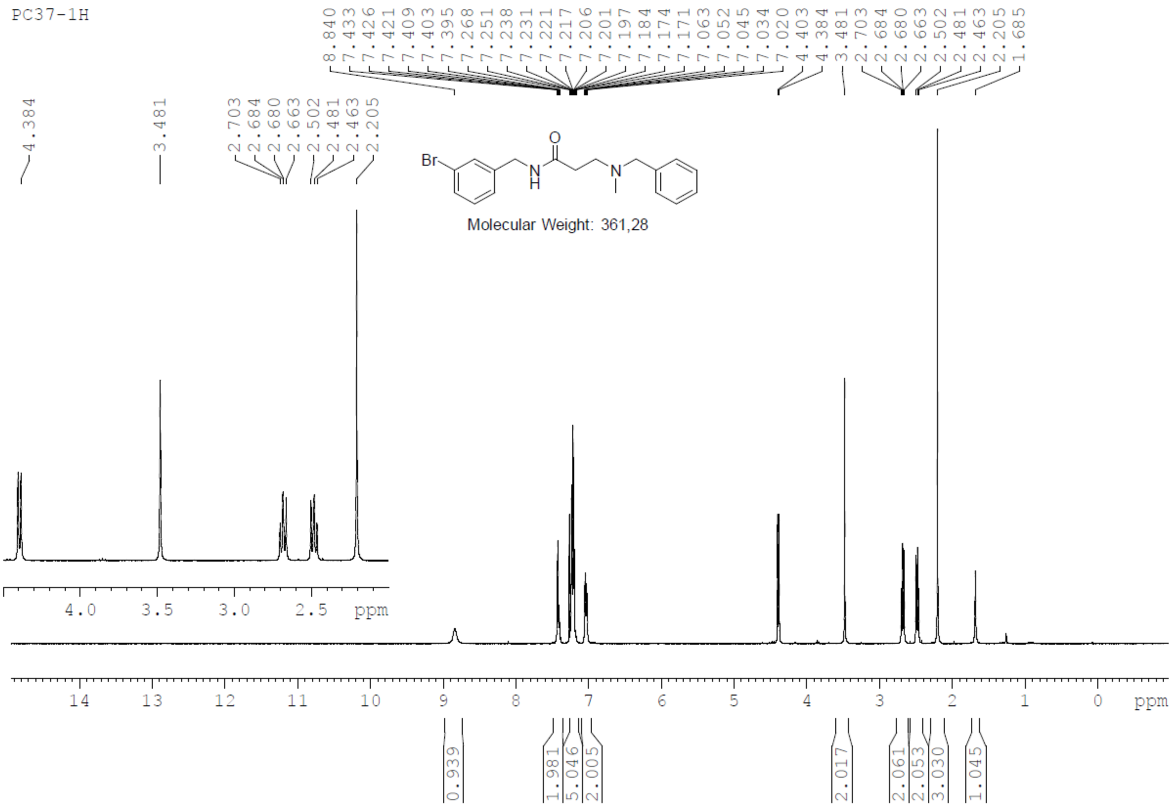

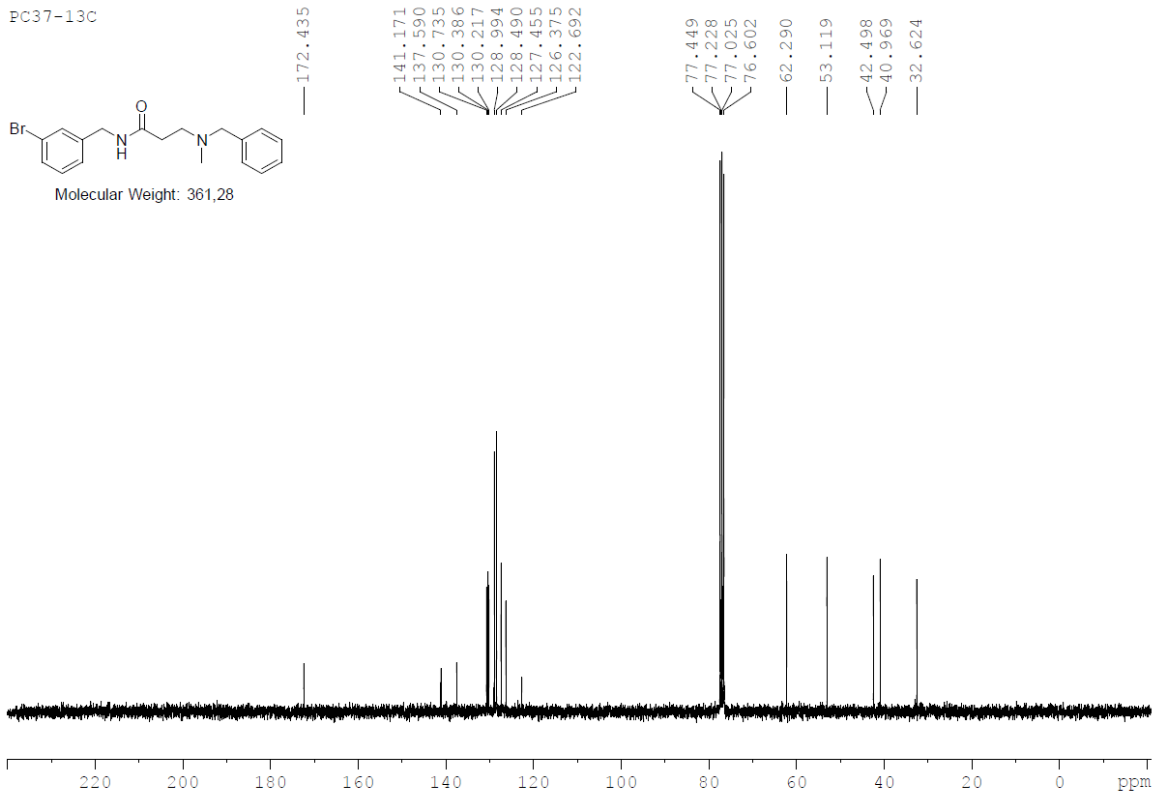

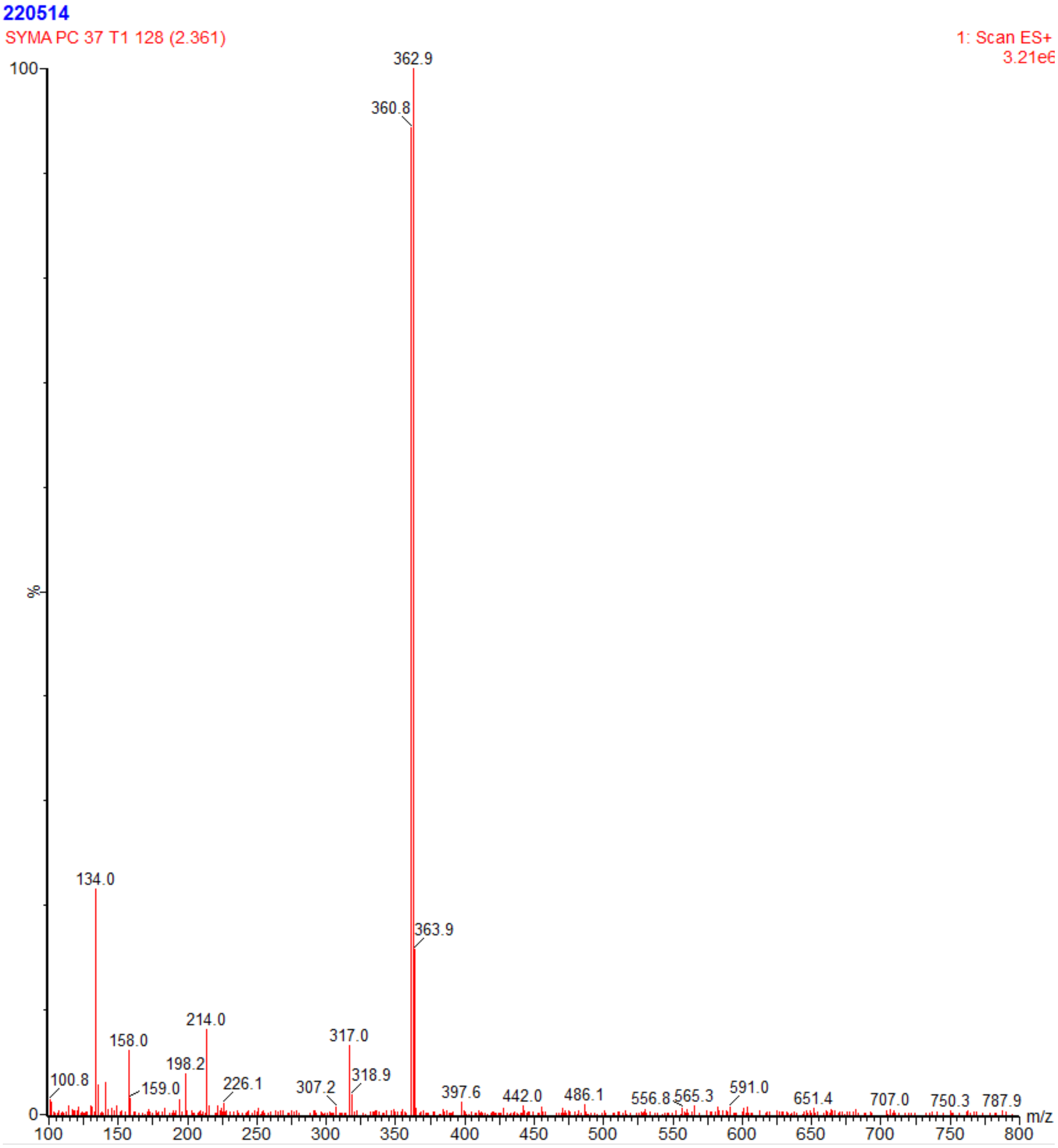
220514
SYMA PC 37 T1 128 (2.361)
1: Scan ES+
3.21e6
362.9
360.8
134.0
363.9
214.0
158.0
317.0
198.2
318.9
100.8
159.0
226.1
307.2
397.6
442.0
486.1
556.8
565.3
591.0
651.4
707.0
750.3
787.9
%
m/z

3-(Benzylmethylamino)-*N*-(2,4-dichlorobenzyl)propanamide (5)

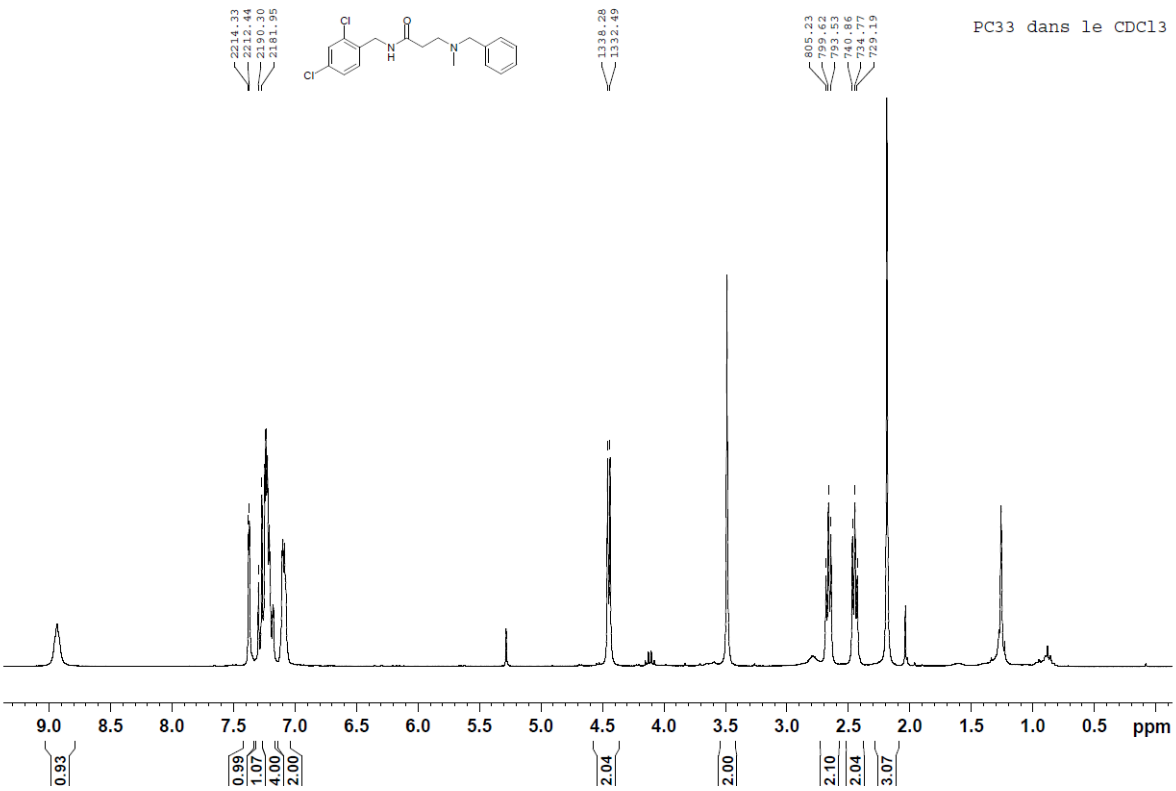

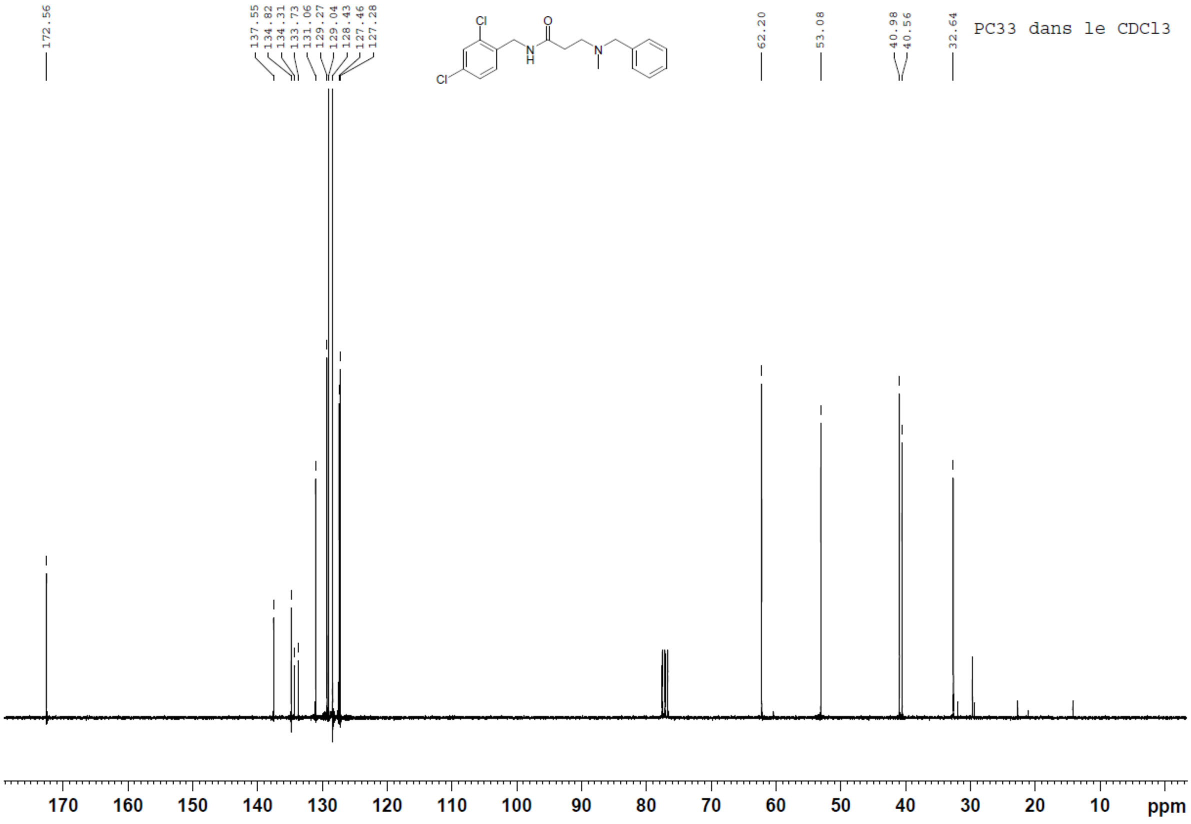

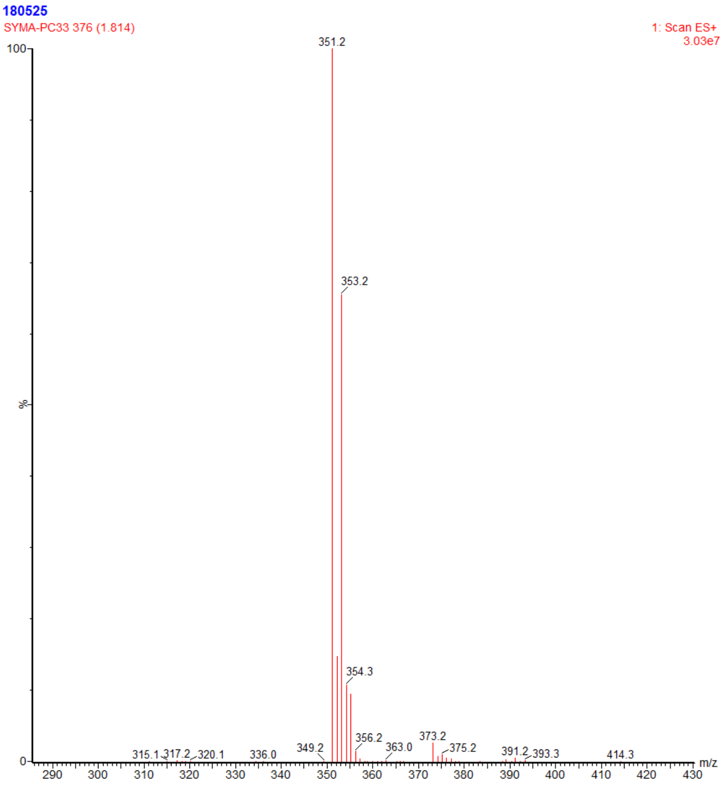
180525
SYMA-PC33 376 (1.814)
1: Scan ES+
3.03e7
100
%
0
351.2
353.2
354.3
356.2
363.0
373.2
375.2
391.2
393.3
414.3
315.1
317.2
320.1
336.0
349.2
290
300
310
320
330
340
350
360
370
380
390
400
410
420
430
m/z

3-(Benzylmethylamino)-*N*-(4-cyanobenzyl)propanamide (6)

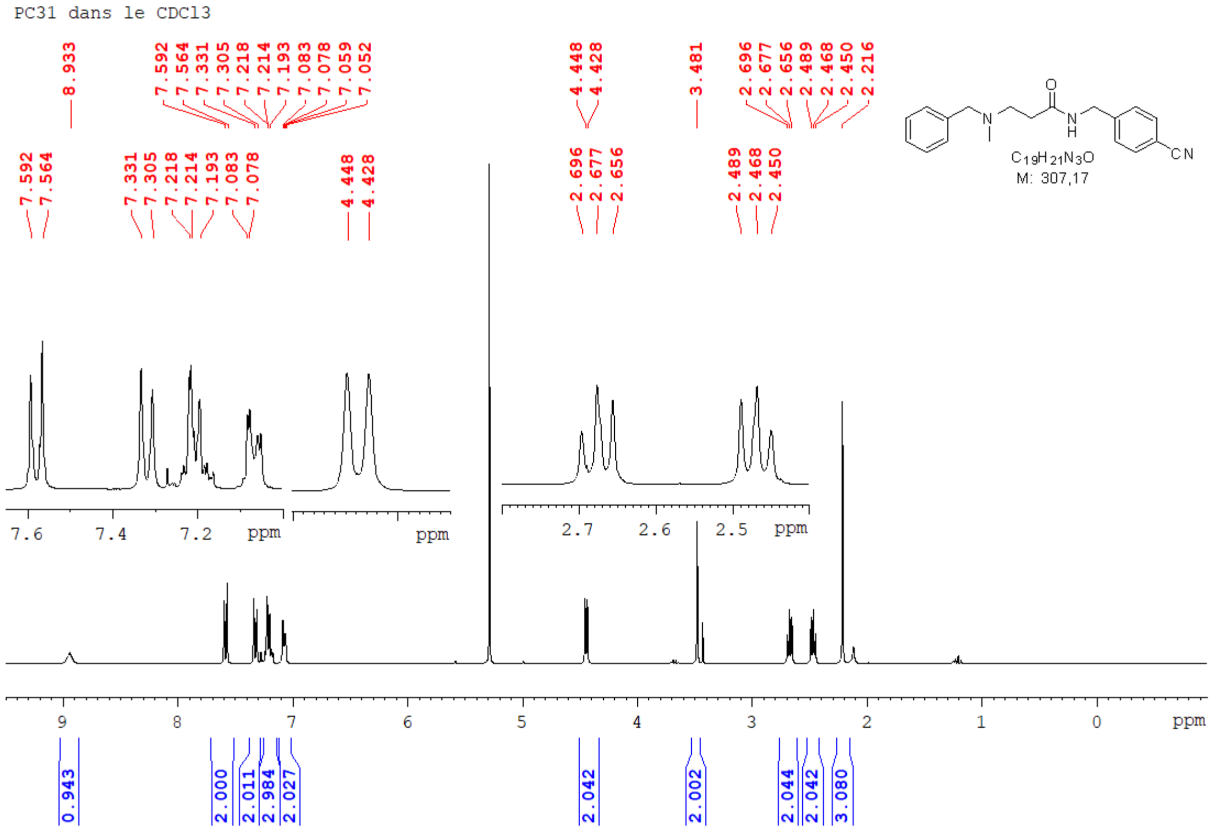

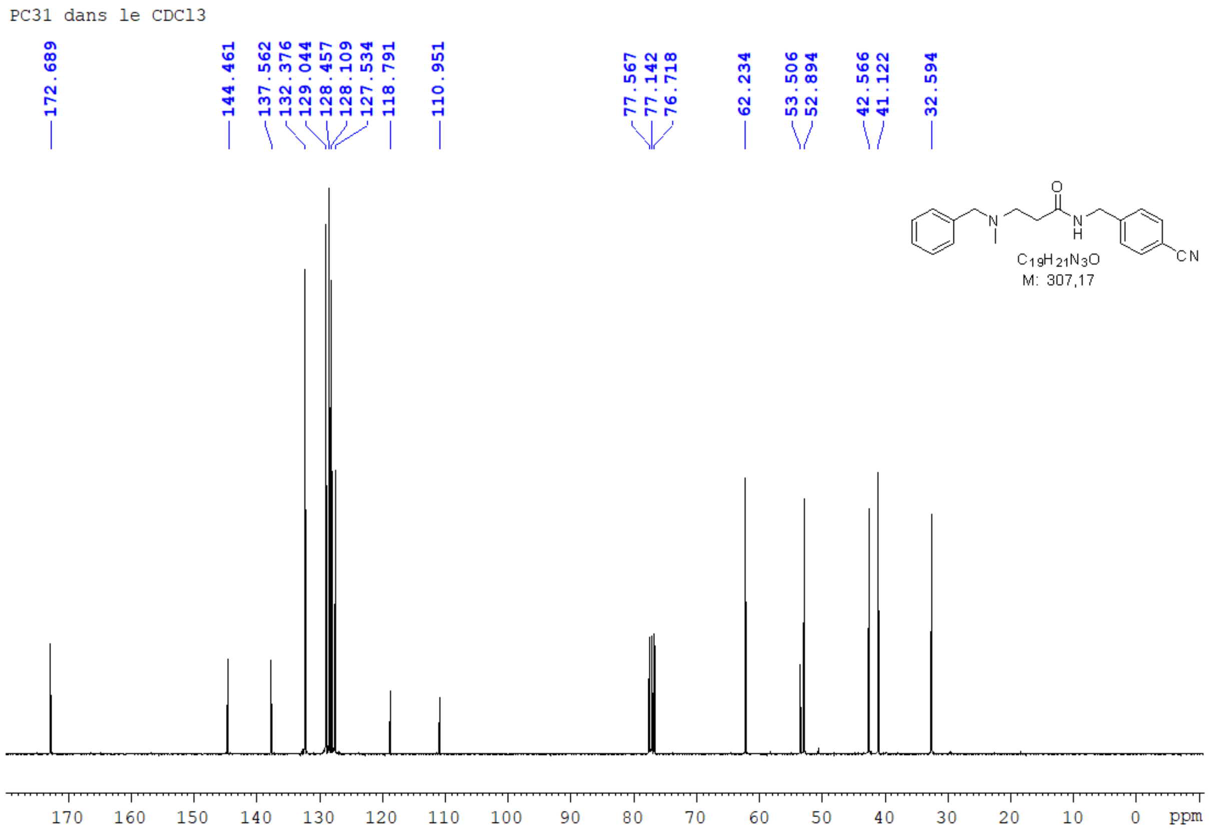

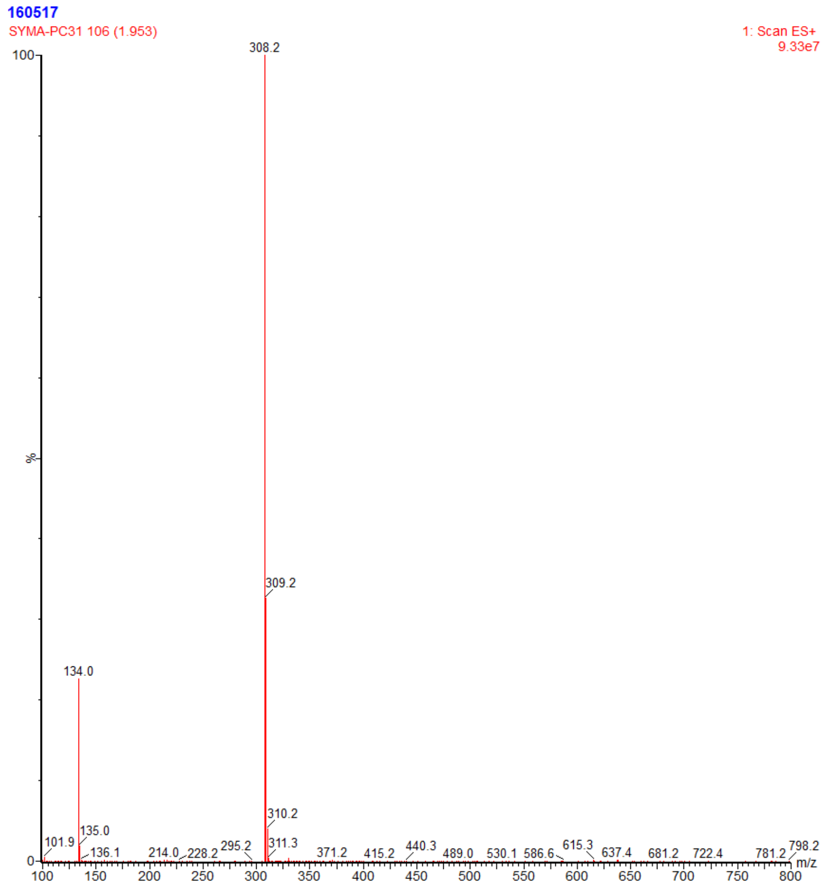
160517
SYMA-PC31 106 (1.953)
1: Scan ES+
9.33e7
308.2
309.2
134.0
310.2
135.0
101.9
136.1
214.0
228.2
295.2
311.3
371.2
415.2
440.3
489.0
530.1
586.6
615.3
637.4
681.2
722.4
781.2
798.2
%
m/z

3-(Benzylmethylamino)-*N*-(4-nitrobenzyl)propanamide (7)

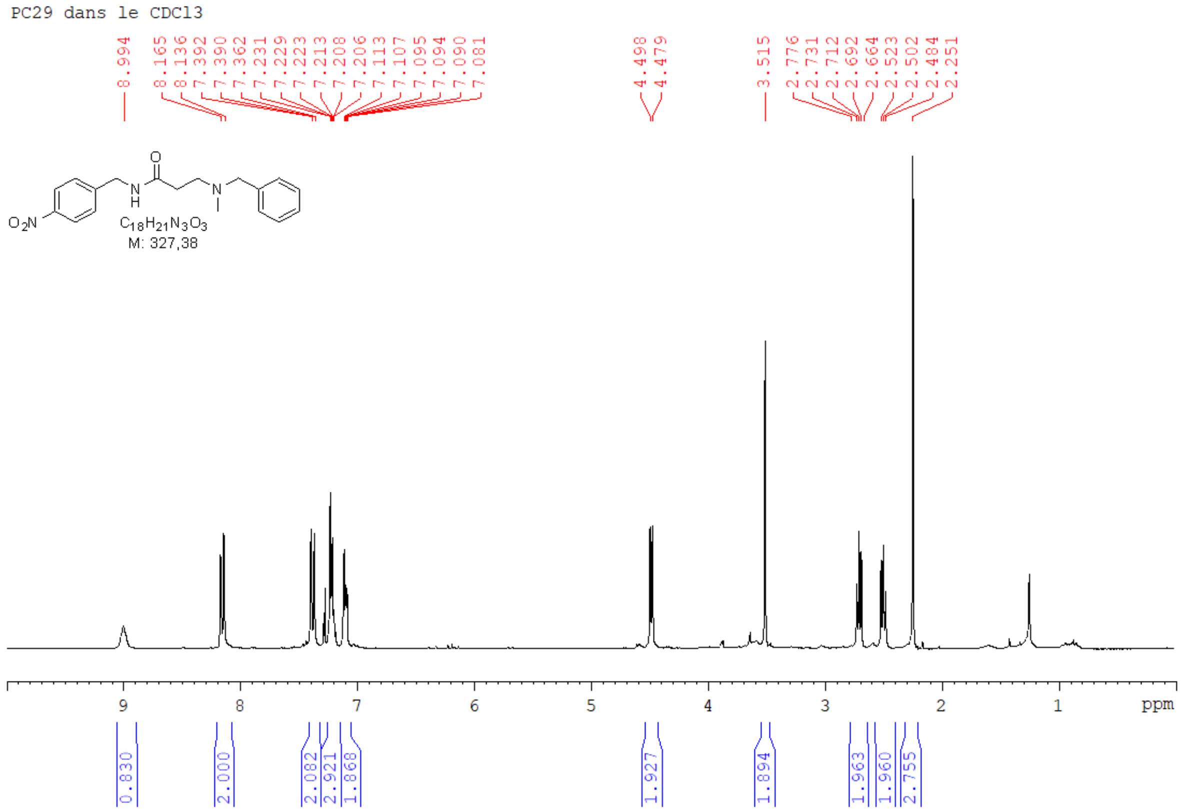

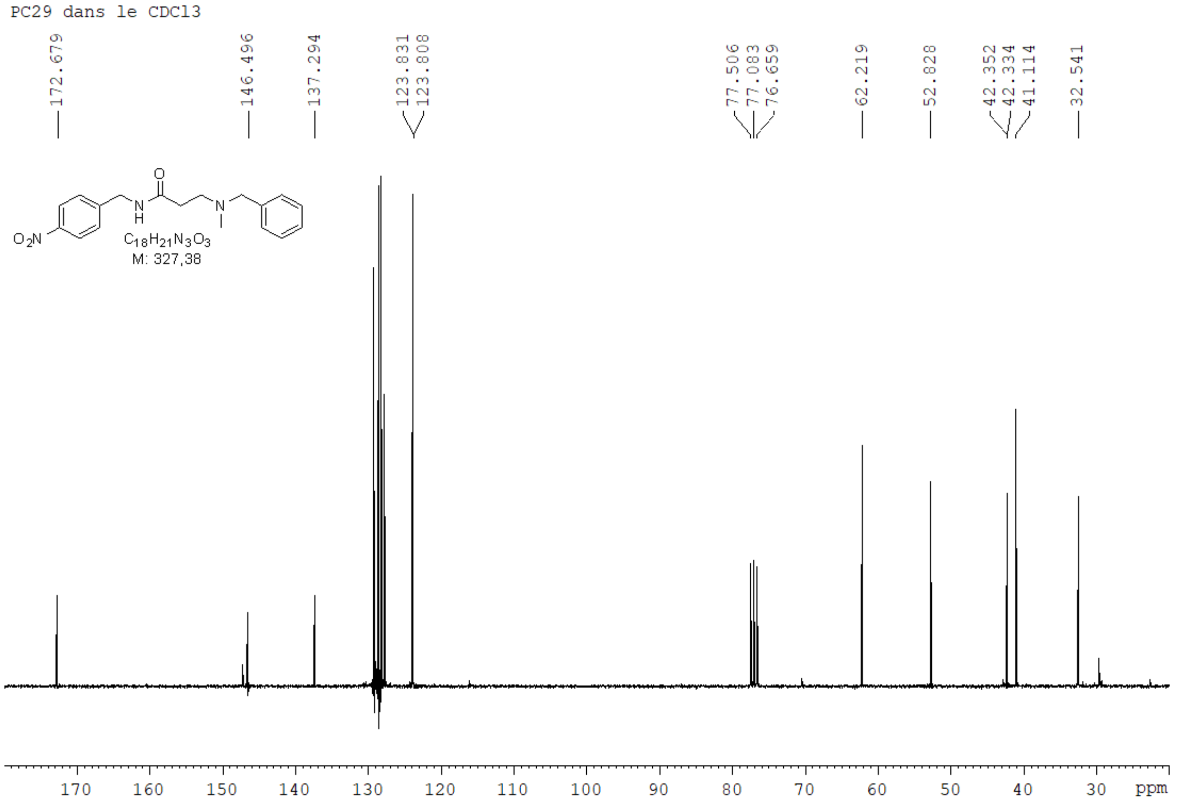

140514

SYMA PC 29 116 (2.139)

1: Scan ES+
8.57e7

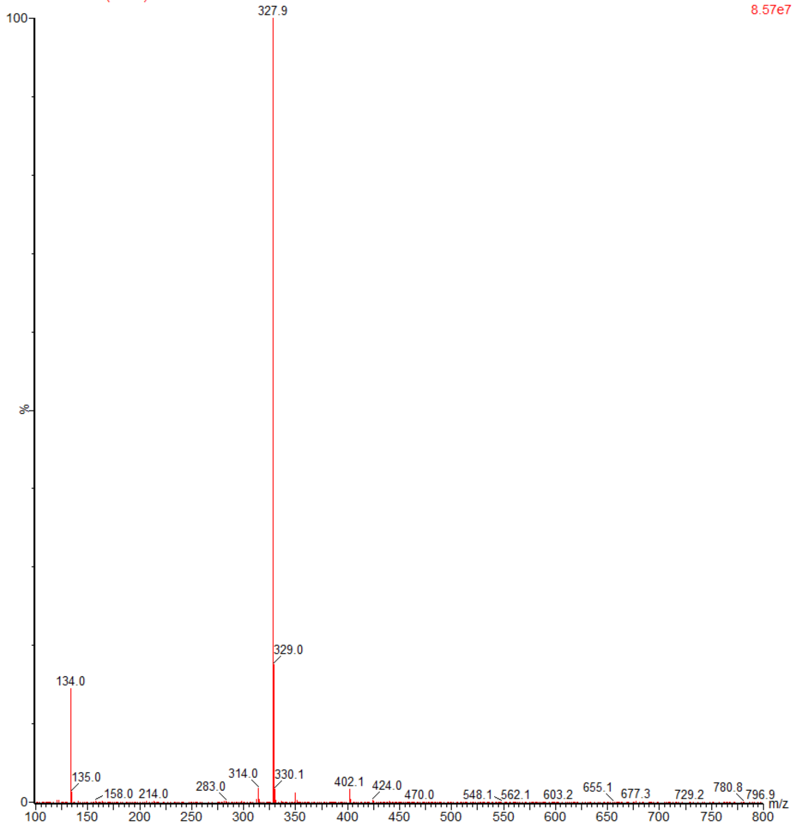

Figure S2. Displacement curves for compounds 1 (a), 2 (b), 3 (c), 5 (d) 6 (e) and 7 (f) against [^{3}H]-(+)-pentazocine binding to S1R sites in Jurkat cell membranes according to Ganapathy ME *et al* [17]. Data are presented as mean ± SEM of n = 2 per condition.

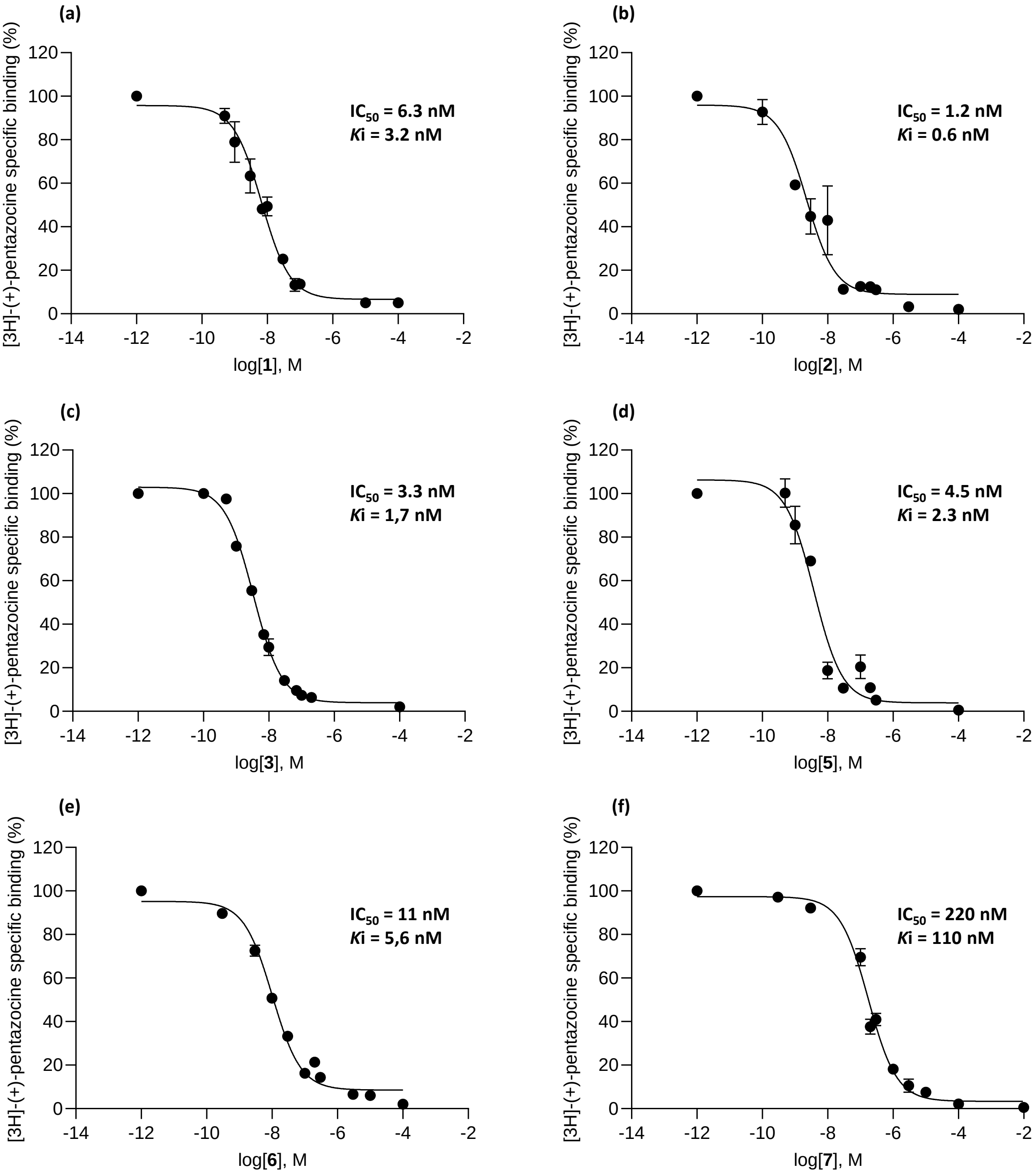

Figure S3. Displacement curves for compounds 1 (a), 2 (b), 3 (c), 5 (d) 6 (e) and 7 (f) against [^{3}H]-(+)-di-o-tolylguanidine (DTG) binding sites in Jurkat cell membranes in the presence of excess of non-tritiated (+)-pentazocine which selectively occupies S1R, according to Ganapathy ME *et al* [17]. Data are presented as mean ± SEM of n = 2 per condition.

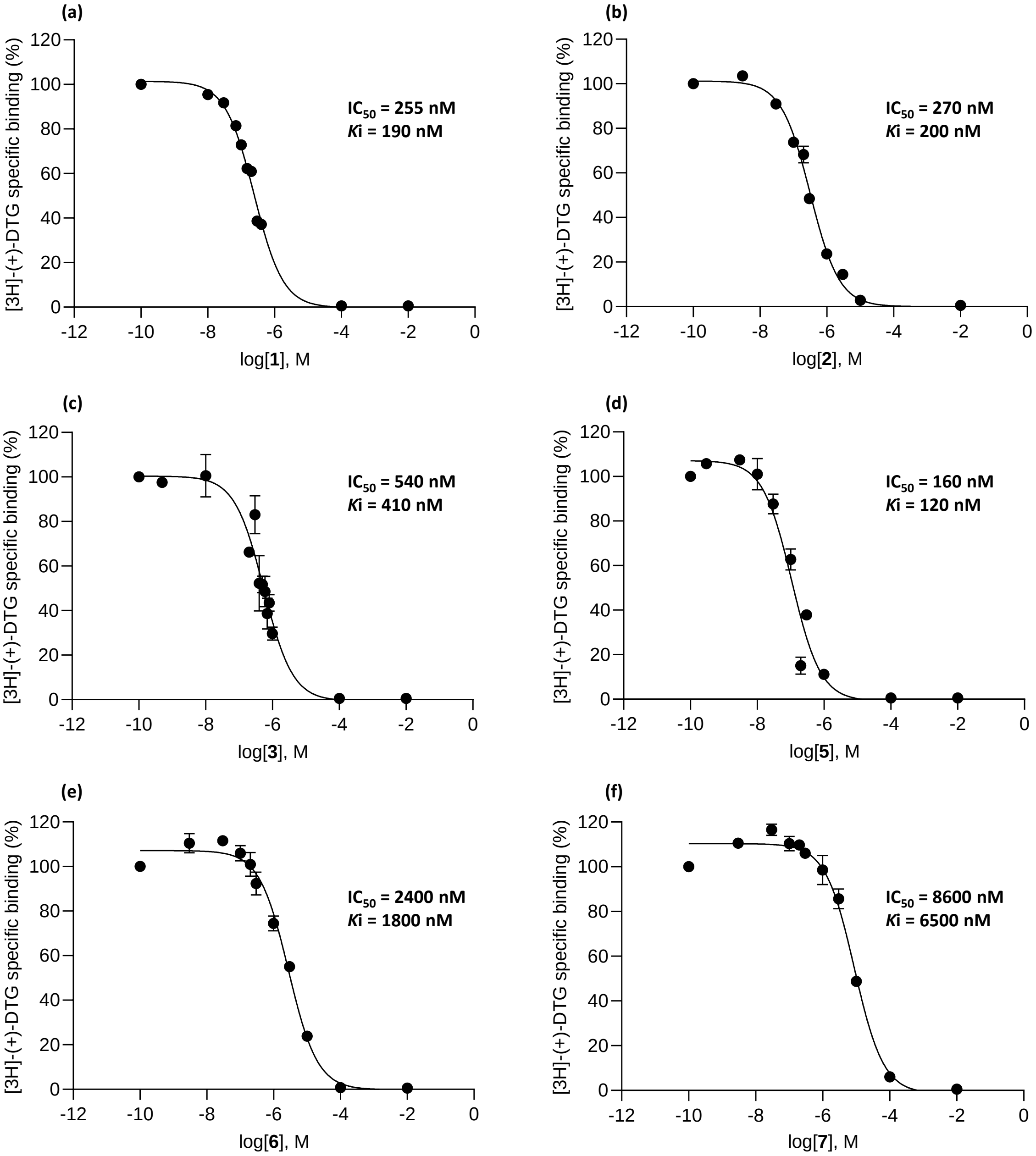

Supplement: Supplementary file 1 [file molecules-30-03584-s001.zip › molecules-3777697-supplementary.pdf]
